# Telomere dynamics in a long-lived bird, the barnacle goose

**DOI:** 10.1186/1471-2148-12-257

**Published:** 2012-12-31

**Authors:** Angela Pauliny, Kjell Larsson, Donald Blomqvist

**Affiliations:** 1 Department of Biological and Environmental Sciences, University of Gothenburg, Box 463, 405 30 Gothenburg, Sweden; 2 Department of Biology, Gotland University, 621 67, Visby, Sweden; 3 Kalmar Maritime Academy, Linnaeus University, 391 82, Kalmar, Sweden

**Keywords:** Individual telomere rate of change, Longitudinal data, Rate of ageing, Senescence, Survival, Telomere maintenance

## Abstract

**Background:**

Theories of ageing predict a trade-off between metabolism, reproduction, and maintenance. Species with low investment in early reproduction are thus expected to be able to evolve more efficient maintenance and repair mechanisms, allowing for a longer potential life span (intrinsic longevity). The erosion of telomeres, the protective caps at the ends of linear chromosomes, plays an important role in cellular and organismal senescence, signalling the onset of age-related disease due to accumulation of unrepaired somatic damage. Using extensive longitudinal data from a long-term study of a natural population of barnacle geese *Branta leucopsis*, we investigated individual rates of telomere length changes over two years in 34 birds between 0 and 22 years of age, covering almost 80% of the species’ lifespan.

**Results:**

We show that telomeres in this long-lived bird are very well maintained, as theoretically expected, with an average loss rate of only 5 base pairs per year among adults. We thus found no significant relationship between change in telomere length and age. However, telomeres tended to shorten at a faster pace in juveniles compared to adults. For the first time, we demonstrate a faster telomere attrition rate in females compared to males. We found no correlation between telomere loss rate and adult survival or change in body mass.

**Conclusions:**

Our results add further support for a link between longevity and telomere maintenance, and highlight the complexities of telomere dynamics in natural populations.

## Background

The evolutionary basis for senescence is one of the major questions in biology. If our germ line is immortal, why do we have to age and die? After half a century of research, we are now beginning to understand better the processes of ageing, the underlying biological mechanisms, and why species have evolved different rates of ageing and lifespans [1]. An organism needs to allocate the finite amount of energy resources to three main tasks: basic metabolism, sex and reproduction, as well as maintenance and repair. The process of ageing commences at the onset of reproduction, as only a smaller proportion of the limited energy resources can then be invested into maintenance and repair. This trade-off between reproduction and maintenance is known as the disposable soma theory of ageing [2], and it helps to explain why species with high levels of extrinsic mortality invest more in early reproduction at the expense of a longer lifespan (due to neglected repair of the soma). Conversely, slower maturation and lower fecundity is favoured in species with high juvenile survival rates, permitting investment in more efficient maintenance mechanisms and hence longer intrinsic lifespans. Ageing can thus be viewed as the result of the accumulation of unrepaired somatic damage [2].

An organism relies on an extensive suite of maintenance mechanisms including DNA repair, protein repair or removal, defences against free oxygen radicals, apoptosis, immune response, and wound healing [1]. In particular, the maintenance of telomeres has emerged as an important factor influencing the rate of organismal senescence. These dynamic nucleoprotein structures at the end of eukaryotic chromosomes have a multitude of vital functions. For example, a telomere and its associated shelterin complex protect the chromosomal end from being recognized as a double-stranded break by the DNA damage responses machinery and thereby promote genome stability [3]. In addition, telomeres ensure correct mitotic separation of sister chromatids [4], and play an important role in modifying the expression of subtelomeric genes [5]. Since only sufficiently long telomeres are able to exert their functions, cells may express the enzyme telomerase to counteract various telomere-shortening factors, including the end-replication problem and oxidative stress [6]. In line with the free radical theory of ageing [7] and empirical studies (e.g. [8], reviewed in [9]), individuals vary in their rate of ageing and thus lifespan depending on their exposure or ability to resist such telomere-shortening factors. Also, differences in maximum longevity among species appear to be correlated with the rate of telomere erosion. Longer-lived birds and mammals were shown to lose telomere repeats at a slower pace than shorter-lived species (e.g. [10,11]), suggesting more efficient maintenance mechanisms in long-lived species.

Telomere length (hereafter TL) and its relationship with age has been thoroughly examined in natural populations of birds, probably because a relatively large number of avian studies have data on marked, known-age individuals. The majority of these studies present cross-sectional analyses, comparing the lengths of telomeres in different individuals of varying ages. However, conclusions about telomere attrition rates from such data have to be treated with caution. There are a number of factors contributing to the large inter-individual variation in TL that are independent of age, potentially confounding inferences about loss rates. First, initial TL is largely heritable in humans, birds and lizards [12-14]. Second, previous studies have demonstrated substantial individual differences in telomere attrition rates (e.g. [11,15]). Third, avian genomes often harbour copious amounts of telomere-like sequences at interstitial locations on the chromosomes, which do not shorten over time but vary significantly among individuals [16]. Fourth, relative measures of TL (corrected for age) correlate with fitness components including lifespan (e.g. [11,15,17]). Thus, preservation or even increase of TL with age in a cross-sectional study may just be the result of selective mortality. To obtain a reliable measure of telomere attrition rate, repeated sampling of the same individual over time is required. There are still few studies of natural populations presenting such longitudinal data, and the available data is limited to either small sample sizes (e.g. [11,17]) or covering only parts of a species lifespan (e.g. [15]).

Here, we present a longitudinal study of telomere dynamics in a long-lived bird species, the barnacle goose *Branta leucopsis*. Building on extensive long-term data, we analyse repeated measurements of blood TL in 34 individuals, ranging from 0–22 years of age, which cover almost 80% of the maximum recorded lifespan of the species. We examine whether the telomere attrition rate correlates with age and predicts fitness parameters such as adult body mass and survival. Finally, given the sex differences in telomere length reported in several taxa (reviewed in [18]) we test whether the rate of telomere erosion differs between the sexes in the barnacle goose.

## Methods

### Study species

The barnacle goose is a migratory long-lived species that mainly breeds in arctic regions of northern Russia, on Svalbard and on Greenland. Since the 1970s, the species has expanded its breeding range within the Arctic, as well as into temperate areas in the Baltic region and in the Netherlands [19-22]. The world population has increased dramatically in both the arctic and temperate breeding areas from about 30 000 individuals in 1959/60 [23] to about 870 000 birds in 2009 [24]. Several research groups have performed long-term studies of breeding barnacle geese in the Baltic region during the past 30 years, focussing on population dynamics, behaviour and genetics (e.g. [21,22,25]). As part of these studies, several thousand juvenile and adult barnacle geese have been marked with metal and individually engraved plastic colour rings in the main Baltic colonies between 1984 and 2012.

### Study population and sampling methods

In mid-July each year, before juveniles are able to fly and when moulting adults are flightless, flocks of barnacle geese were captured on the moulting grounds close to the main breeding colonies on Laus holmar off the east coast of Gotland, Sweden (57°17´ N, 18°45´ E), using a rounding-up technique. At capture, we discriminated between juveniles, i.e. 6 to 8 weeks old birds, and adults, i.e. one-year old or older birds. We determined the sex of captured birds by cloacal examination [26]. Individual body mass was measured to the nearest 25 g with a Pesola spring balance. The exact age of recaptured adults in specific years could be determined for birds that had previously been ringed as juveniles. The oldest individual in the study population was observed alive when it was at least 26 years old. The recorded maximum age of a marked bird in the Svalbard barnacle goose population is 28 years. Mean adult annual survival rates in the study population between 1984 and 1996 have previously been estimated to approximately 96% for females and 95% for males [27].

During the capture procedures in year 2006 and 2008, we sampled approximately 100 μl blood from the wing vein of captured birds. Blood sampling followed national legislations and was performed under ethical licences Dnr. 8–06 (KL through J. Waldenström, Linnaeus University, Sweden) and 259–2008 (DB). The blood was mixed with SET-buffer in the field and stored at −20°C until further analyses.

### Determination of telomere length and rate of change

Telomere restriction fragments were prepared as previously described [11,14]. In brief, high molecular weight genomic DNA was carefully prepared from whole blood using proteinase K digestion at 37°C and standard phenol/chloroform extraction [28]. We digested 5 μg of genomic DNA with *Hae*III and size-separated telomere fragments on a 0.6% non-denaturing agarose gel for 23 hours at 2V/cm using constant-field gel electrophoresis. All individuals were randomly assigned to one of four gels and pairs of repeat samples were analysed in adjacent lanes for optimal comparability. Two λ/*Hind*III size markers were placed at each end to assess uniformity of DNA migration across the gel. After standard Southern blotting [28], telomere fragments were hybridized to an alkaline phosphatase-linked telomere-specific probe and detected by chemiluminescence using CDP-Star (1,2-dioxetane) as a substrate (AlkPhos labelling and detection kit, GE Healthcare).

Digitalized signals were analysed as previously described [11]. The upper limit of the window was set to include the distinct start of the smear, corresponding to the longest telomere fragments at approximately 30 kb. Since there was no obvious fading of the smear, the lower limit was selected to coincide with the shortest size standard fragment (2 kb) available. The analysis of telomere fragments with TELOMETRIC complied with all relevant conditions (e.g. type of electrophoresis and window of analysis) for which the program has been validated ([29]; M. Ochs, personal communications). Although this program and its interpolation algorithm had been questioned by some in the past (e.g. [30]), recent studies comparing TELOMETRIC with another imaging software, ImageJ, clearly demonstrated the validity of results for within-species comparisons with either program [14,17]. A representative picture of telomeric profiles is shown in Additional file 1: Figure S1. In contrast to dunlins *Calidris alpina* (a shorebird; A. Pauliny, unpublished observations), barnacle geese appear to have abundant interstitial regions. These telomere-like sequences are located within chromosomes and are therefore unaffected by telomere-shortening processes such as ageing [16], as also documented here (see Additional file 1: Figure S1). As the focus of the present study is to investigate changes in telomere length within the individual, however, the presence of such interstitial sequences does not interfere with our analyses.

In total, we determined TL in 80 known-age samples of barnacle geese. Thirty-four individuals (30 adults and 4 juveniles) were sampled twice (in 2006 as well as in 2008). Their telomere rate of change (TROC hereafter) over this two year period was calculated as the difference in TL between year 2008 and 2006, with negative values denoting a decrease in length. In addition, we obtained single TL measurements from 4 and 8 juveniles born in 2006 and 2008, respectively. The ages of individuals in this dataset covered most of the species’ lifespan, ranging from 0 to 22 year old birds. The total number of 46 individuals analysed included 18 males and 28 females.

### Statistical analyses

For descriptive purposes, we present means ± standard errors. As found in previous studies [11], TL measurement were not normally distributed (mean TL: Shapiro-Wilks test, W = 0.92, p = 0.01, n = 34). We therefore used non-parametric statistical tests throughout the paper. Differences between means were examined with randomization tests (e.g. [31], as implemented by the software RESAMPLING STATS), using the t-variable as the test statistics and running 10 000 iterations.

Some studies have reported a correlation between attrition rate and telomere length at first sampling (e.g. [32]). However, we found no significant effect of TL in 2006 on TROC (measured as proportional change) in adult barnacle geese (Spearman rank correlation, r_s_ = −0.14, p = 0.47, n = 30).

## Results

**Figure 1:**
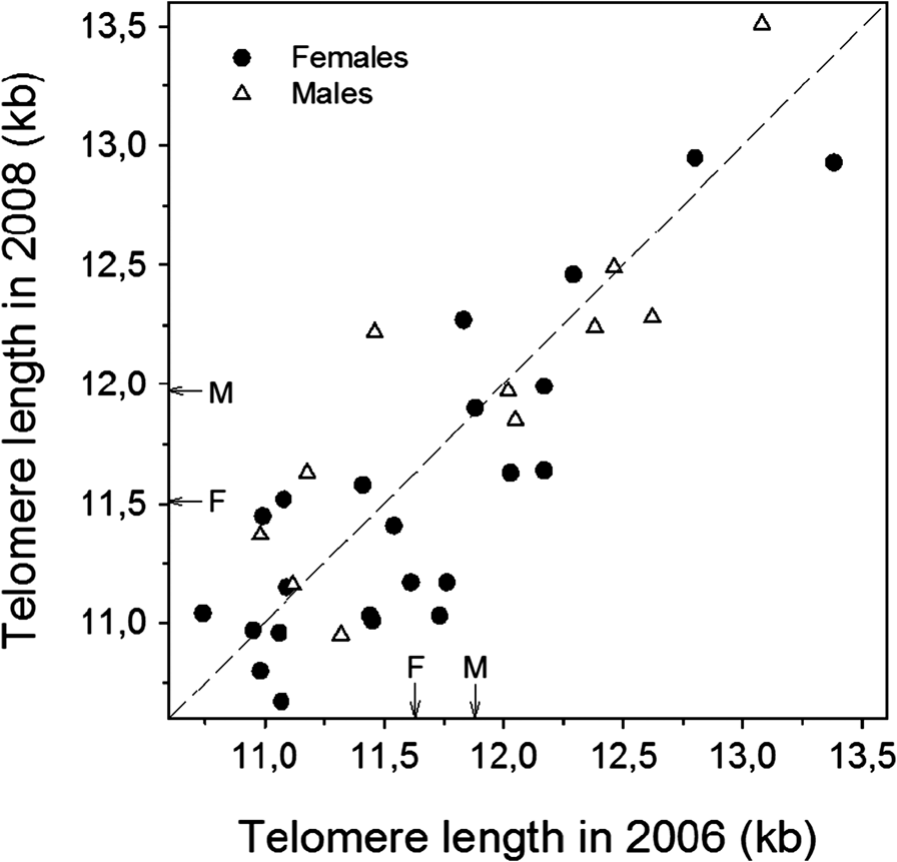
**Repeat measurements of telomere length (TL) in barnacle geese. **Estimates of TL (in kb, kilobases) from the same individual, sampled in 2006 and 2008, were highly repeatable within individuals (Spearman rank correlation, r_s_ = 0.82, p < 0.001, n = 34). Females (filled circles) and males (open triangles) are depicted separately. The dashed reference line indicates no change in TL, whereas arrows labelled with F (female) and M (male) denote mean values of TL for each sex in that year.

Pooling all measurements, TL in barnacle geese ranged from 10.67 kb to 13.51 kb (mean ± SE: 11.73 ± 0.07 kb; n = 80). Repeated sampling of 34 individually marked geese in year 2006 and 2008 showed that TL measurements were highly repeatable within individuals (p < 0.001; Figure 1). Most of the observed variation in TL was therefore due to differences among individuals and only a smaller proportion was due to differences between years, age and other factors.

### Longitudinal analyses of telomere attrition rate

**Figure 2:**
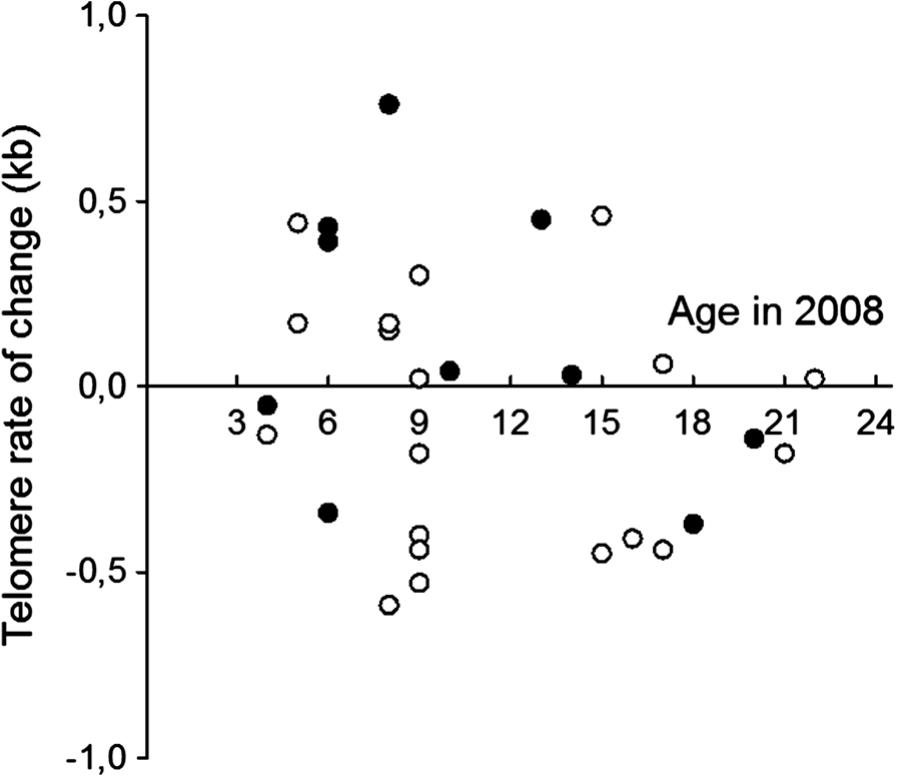
**Longitudinal analysis of telomere rate of change in adult barnacle geese. **Our assays indicated both increases and decreases in telomere length among adult birds (2 years or older at first sampling). The relationship between these changes (irrespective of direction) and age at second sampling was non-significant (Spearman rank correlation on absolute values, r_s _= −0.09, p = 0.63, n = 30). A negative value denotes a shortening of telomeres between 2006 and 2008. Data for males (filled circles) and females (open circles) are shown separately.

Since cross-sectional analyses may not accurately reflect changes within individuals over time, for example due to selective mortality, we performed longitudinal analyses. Among adults (2 years or older at first sampling), we detected only relatively small changes in TL, including both increases and decreases (Figure 2). We therefore tested whether TROC correlated with age, independent of the direction of change. This analysis, based on absolute values of TROC, revealed no significant relationship with age (p = 0.63; Figure 2), implying that telomeres are well maintained in adults. The correlation was non-significant also when we used the original TROC values in the analysis (not shown).

**Figure 3:**
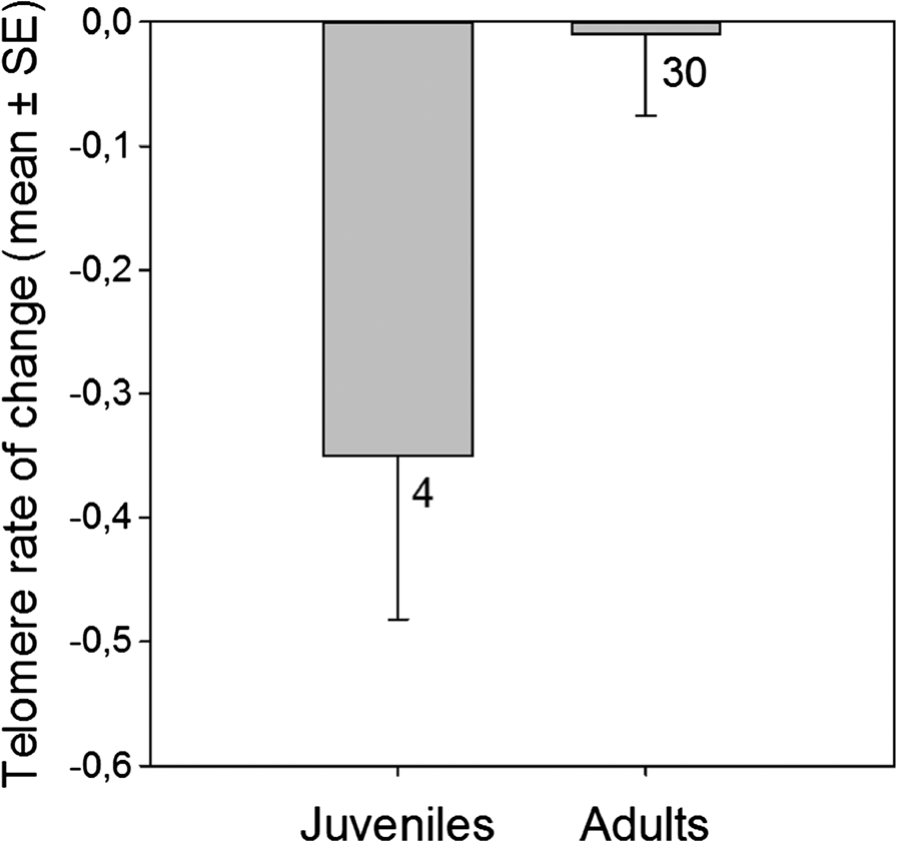
**Mean telomere rate of change (in kb) in juvenile and adult barnacle geese. **During a 2-year period, juvenile birds tended to lose telomere repeats faster than adults (Randomization test, t = 1.66, p = 0.07). Numbers below bars denote sample sizes.

Many previous studies, including work on long-lived birds (e.g. [11]), have shown that telomere shortening over time may be non-linear, with faster attrition rates in early life stages. We therefore compared TROC in juvenile birds (6–8 weeks old at first sampling) with those of adult birds. All four juveniles lost telomere repeats during the 2-year period, whereas such losses were only recorded in about 50% of the adults (see Figure 2). Comparing mean TROC, we found that telomeres shortened at a faster pace in juveniles than in adults, albeit not significant at the 5% level (p = 0.07; Figure 3).

### Correlates of telomere attrition rate

**Figure 4:**
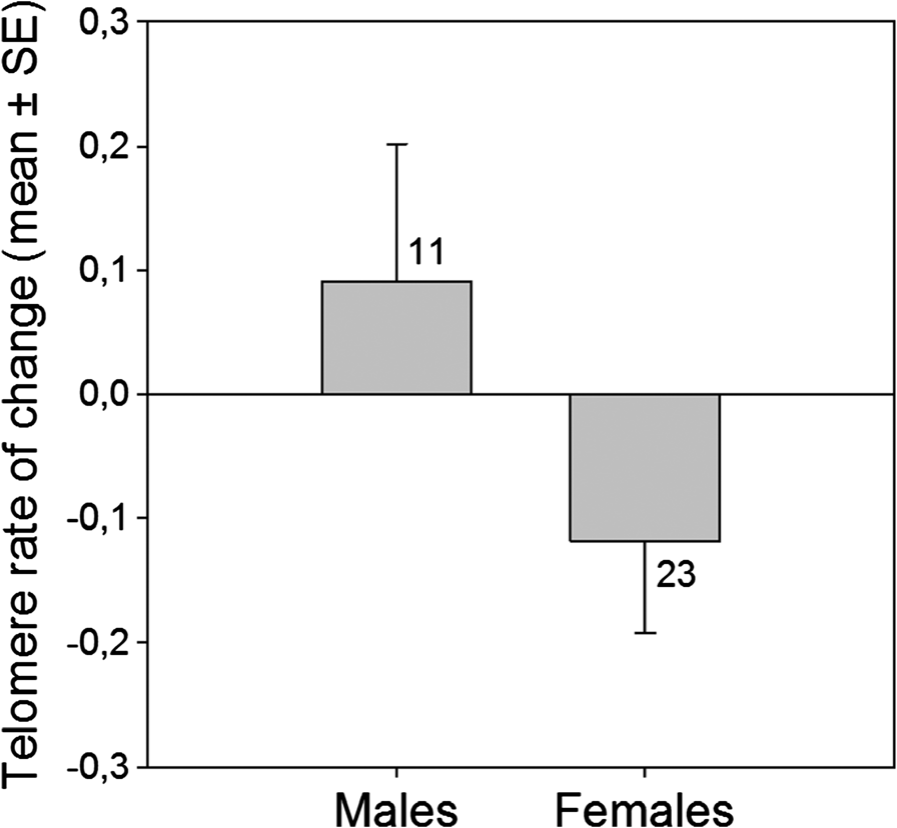
**Mean telomere rate of change (in kb) in male and female barnacle geese. **Females lost telomere repeats at a faster rate than males (Randomization test, t = 4.52, p = 0.03). Numbers above and below bars denote sample sizes.

Individual TROC differed between the sexes: female barnacle geese lost telomere repeats more rapidly than males (p = 0.03; Figure 4, cf. Figure 1 for differences in TL).

TL and TROC have previously been shown to correlate with fitness in other bird species (e.g. [11,15]). We therefore tested the predictions that individuals who maintained their telomeres better were in better condition or had higher future chances of survival. However, we found no significant correlation between individual TROC and change in body mass of adult birds between 2006 and 2008 (Spearman rank correlation, r_s_ = 0.11, p = 0.56, n = 29). Of the 30 adults that were captured and analysed in 2006 and 2008, 23 birds were recaptured on moulting sites in 2009 or later. Mean TROC did not differ significantly between adults that were known to be alive in 2009 (0.01 ± 0.08, n = 23) and those that were not recaptured in 2009 or later (−0.001 ± 0.14, n = 7; Randomization test, t = −0.10, p = 0.92). Thus, we found no indication that the change in TL in adult birds was correlated with a change in condition (measured as body mass during moult) or their survival between 2008 and 2009.

## Discussion

Our long-term dataset on a wild population of barnacle geese allowed us to examine telomere dynamics on an individual basis, covering almost 80% of the lifespan of this long-lived species. Using this longitudinal dataset, we demonstrate that within each individual, telomeres are well maintained throughout life. Thus, our study adds further empirical support to theories of ageing, predicting a link between efficient maintenance mechanisms and organismal longevity [1,2].

Previous studies based on cross-sectional estimates of TROC in birds concluded that telomeres of long-lived species are lost at a slower pace than those of short-lived species [10,11]. By large, this pattern seems to hold true even in mammals (see [10]). Slower telomere attrition rates may be explained by either slower production rates of oxygen radicals [33] or lower levels of oxidative damage in long-lived mammals [34], perhaps indicating a more efficient repair mechanism. Telomerase, a reverse transcriptase able to *de novo* synthesize telomeric DNA, represents one such mechanism to restore telomeres [35]. Indeed, in two relatively long-lived bird species telomerase activity is maintained even in adult somatic cells, whereas this is not the case in two bird species with shorter life spans [36].

In adult barnacle geese, we found no significant relationship between telomere rate of change and age. Overall, individual TROC in this long-lived species seems negligibly small, with an average loss rate of only 5 bp per year among adults (25 bp per year if juveniles are included). Telomeres shortened by replicative senescence or oxidative stress damage thus seem to get restored. The observed rate in the present study is in accordance with predictions for TROC given the maximum recorded age of 28 years in this species, and is comparable to data from a cross-sectional study in the common tern *Sterna hirundo* (maximum observed lifespan 26 years, [10]).

A cross-sectional analysis of TL (including potentially interfering interstitial sequences) corroborates our results based on the longitudinal data. We thus found no correlation between TL and age of individuals (data not shown), as has been documented in other wild populations of long-lived species such as the kakapo *Strigops habroptilus* (a parrot; [13]). Despite substantial variation among individuals (almost 30%), our estimates of TL from the same individual (taken two years apart) are highly correlated, thus confirming the validity of our method for measuring TL.

Compared to adults, young barnacle geese tended to lose telomere repeats at a faster rate. Indeed, mean attrition rate was on average 35 times higher in juveniles (Figure 3), though not significant at the 5% level (only four juveniles sampled). Some previous studies have also reported a non-linear decline of TL with age, but these results were not based on longitudinal data (e.g. [37]).

Several studies have documented sex differences in TL and, assuming no difference at birth, often inferred that the attrition rate differs between the sexes (reviewed in [18]). However, most of these studies are based on cross-sectional data and, therefore, do not provide direct evidence for a difference in individual TROC between males and females (but see [38] and [39] for faster attrition rates in males in some circumstances). To the best of our knowledge (see [18]), we show for the first time that individual telomere attrition rate is higher in females compared to males. There are many hypotheses trying to explain the underlying mechanisms for sex-specificity in TL and loss rate, and several indeed predict a higher telomere attrition rate in the heterogametic sex; i.e. females in birds (reviewed by [18], see also [13],[40]). One previous study on birds found shorter telomeres in adult female kakapos [13], consistent with our data on sex differences in TL in the barnacle goose (see Figure 1).

Estimates of TROC as well as relative measures of TL have been shown to correlate with lifespan [11,17] or probability of survival to the following year (e.g. [15]). Assuming limited variation in TL due to environmental factors (e.g. telomere loss caused by stressful rearing conditions) such correlations could be the result of either having inherited relatively long telomeres, or being able to better repair and maintain one’s telomeres. In the barnacle goose, TROC (or TL, data not shown) did not seem to correlate with adult condition or survival. Given the limited variation in TROC among adult barnacle geese, however, larger sample sizes might be required for detecting such relationships in this species. A previous study examined the relationship between fitness and TL in species with different longevities. In the short-lived sand martin *Riparia riparia*, individuals with relatively long telomeres lived longer, whereas no such relationship was found in the long-lived dunlin [11]. These findings, as well as the present study, corroborate the theoretically expected positive relationship between a species’ somatic maintenance/repair mechanisms (influencing e.g. telomere dynamics) and its intrinsic longevity (e.g. [2]). They are also consistent with empirical studies demonstrating that cultured cells from long-lived birds, when challenged with oxygen radicals, survived longer and exhibited much higher resistance to oxidative damage than cells from short-lived birds [41].

## Conclusions

Our study supports a link between organismal longevity and maintenance of cellular structures such as telomeres, as predicted by theories of ageing and suggested by previous empirical studies [e.g. 10]. Moreover, we show that telomere attrition rate is higher in females. Although theoretically expected and reported in the heterogametic sex of other taxa, this has never been documented before in birds.

## Competing interests

The authors declare that they have no competing interests.

## Authors’ contributions

AP carried out the molecular genetic work, participated in the design of the study and data analyses, as well as drafted the manuscript. KL provided the long-term data, and DB performed the statistical analysis. KL and DB conceived and designed the study, as well as contributed to the writing of the manuscript. All authors read and approved the final manuscript.

## Supplementary Material

Additional file 1 **Figure S1. **A representative picture of telomeric profiles in barnacle geese. Click here for file
