# Supplementary material for: Telomere dynamics in a long-lived bird, the barnacle goose
Source: BMC Evol Biol. 2012 Dec 31;12:257. doi: 10.1186/1471-2148-12-257 (PMC3546850; doi:10.1186/1471-2148-12-257)
Supplement: Additional file 1 — Figure S1. A representative picture of telomeric profiles in barnacle geese. [file 1471-2148-12-257-S1.pdf]

ADDITIONAL FILE 1

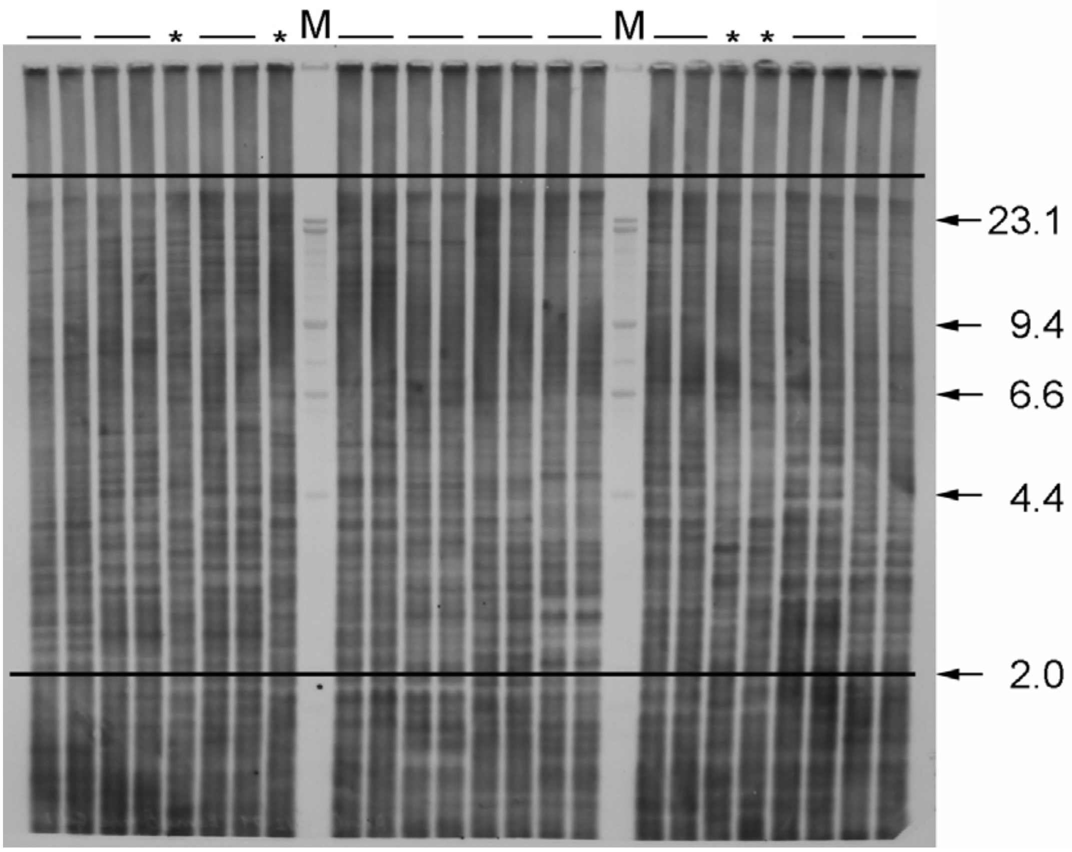

Figure S1 - Telomeric profiles of barnacle geese

Individuals were randomly allocated to one of four gels (one of them depicted here as an example). For optimal comparability, repeat samples were analysed in adjacent lanes (indicated by a dash spanning two wells). Each profile contains abundant insterstitial bands, but note that the banding pattern remains unchanged over time in repeat samples. Asterisk (*) denote samples for which only one point measurement of telomere length was available. Telomere fragments as well as two size standards (M) were size-separated on a non-denaturing agarose gel, and detected by chemoluminescence. The mean length of telomere fragments was estimated within the outlined window using the size standards (fragment sizes presented in kilobases, see Methods for further details).
